# P-151. Cutaneous Larva Migrans Within the United States Military Health System and Association with International Travel

**DOI:** 10.1093/ofid/ofae631.356

**Published:** 2025-01-29

**Authors:** Elena Crecelius, Patrick Hickey, Alison Helfrich

**Affiliations:** Walter Reed National Military Medical Center - National Capital Consortium, University Park, Maryland; Uniformed Services University, Bethesda, Maryland; Uniformed Services University of the Health Sciences, Bethesda, Maryland

## Abstract

**Background:**

Cutaneous larva migrans (CLM) is a parasitic dermatitis which occurs after zoonotic hookworm larva from the soil penetrate the skin. CLM is most common in tropical and sub-tropical regions of the world and in returning travelers from those regions. Within the United States (U.S.) autochthonous cases of CLM have been reported, however the epidemiology is not well-described. We seek to describe the epidemiology of CLM and association with geographic location and international travel within the U.S. military health system.

**Figure:**
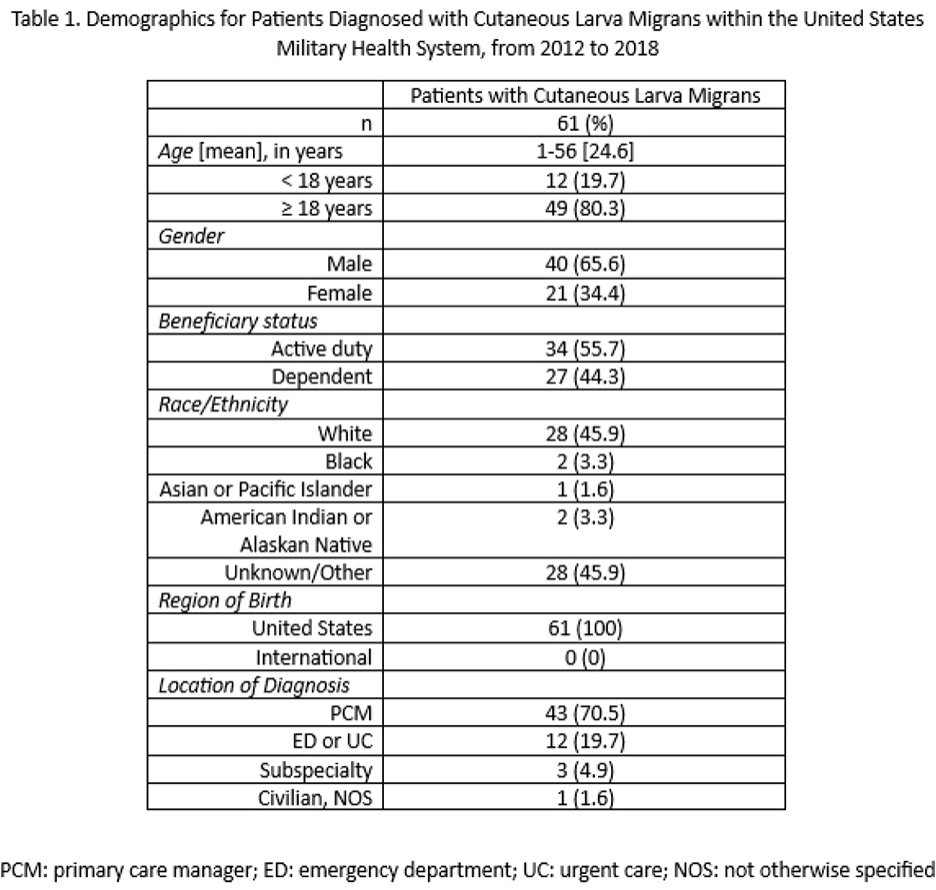

**Methods:**

We performed a retrospective cohort study of active duty service members and their family members who had a diagnosis code of hookworm between October 2012 and September 2018. Of the initial cohort (n = 272), 61 (22.4%) were diagnosed with CLM.

**Figure:**
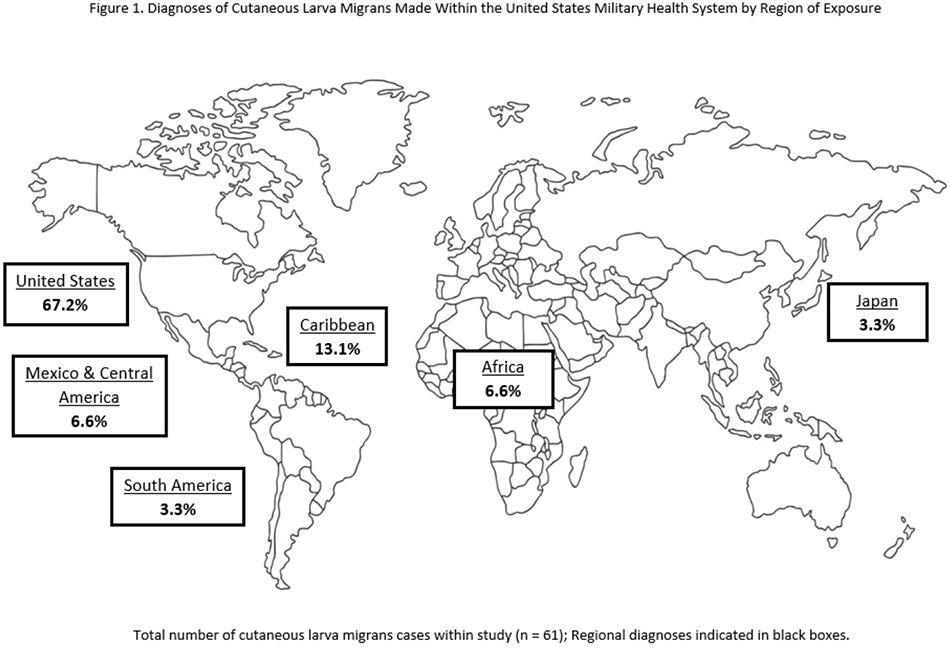

**Results:**

Most patients in this study were male (65.5%), active duty (55.7%), and white (45.9%) [*Table 1*]. Patients’ ages ranged from 1 to 56 years (mean 24.6) with 80.3% of patients 18 years of age or older. All the patients were born within the United States. Most were diagnosed by their primary care manager, however 19.7% were diagnosed via emergency or urgent care. Patients reported preceding travel to tropical and subtropical world regions in 32.8% of cases: Caribbean (13.1%), Africa (6.6%), Mexico and Central America (6.6%), South America (3.3%) and Japan (3.3%) [*Figure 1*]. After excluding those with preceding international travel, 67.2% of patients had autochthonous CLM. Of the autochthonous cases, more patients were diagnosed or had preceding travel in the southeast U.S. (68.3%) compared with the western U.S. (19.5%), southwest U.S. (9.8%), or northeast U.S. (2.4%) [*Figure 2*]. States with the highest number of cases were Florida (26.7%), Georgia (14.6%), North Carolina (9.8%), Texas (9.8%), Washington (7.3%) and Hawaii (7.3%). Based on the reported literature, we found a lower number of cases with preceding international travel than expected. Most autochthonous cases within the U.S. were from the southeast and coastal regions.

**Figure:**
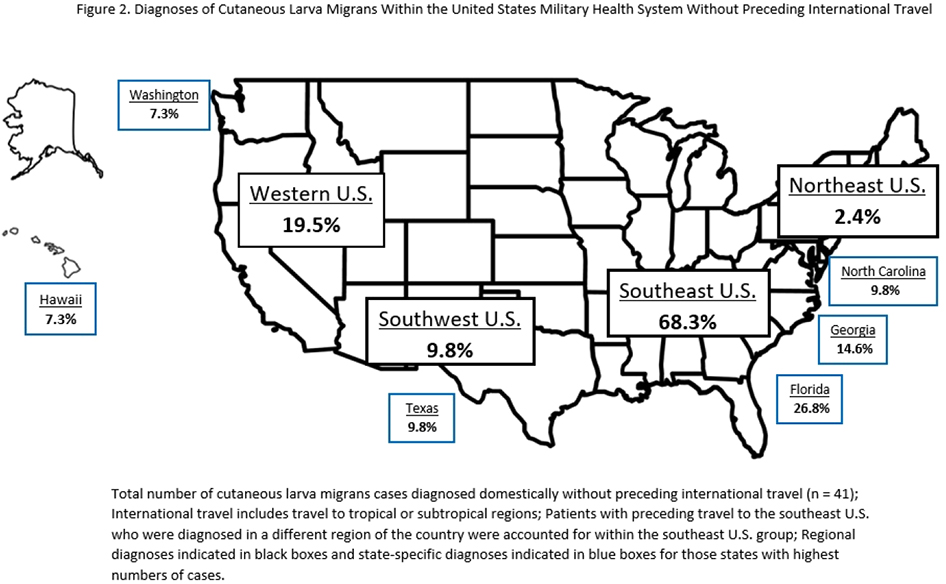

**Conclusion:**

CLM should be considered in the U.S. even in patients without recent international travel. These findings are useful in providing nationwide data on the epidemiology of CLM and allowing for informed preventative counseling to travelers and non-travelers within the U.S.

**Disclosures:**

**Alison Helfrich, DO, MPH**, Moderna: Stocks/Bonds (Public Company)|Sanofi: Stocks/Bonds (Public Company)

